# Potential of *Trichoderma* spp. for Biocontrol of Aflatoxin-Producing *Aspergillus flavus*

**DOI:** 10.3390/toxins14020086

**Published:** 2022-01-23

**Authors:** Xianfeng Ren, Maria Teresa Branà, Miriam Haidukowski, Antonia Gallo, Qi Zhang, Antonio F. Logrieco, Peiwu Li, Shancang Zhao, Claudio Altomare

**Affiliations:** 1 Institute of Agricultural Quality Standards and Testing Technology, Shandong Academy of Agricultural Sciences, Jinan 250100, China; renxianfenga@163.com; 2 Shandong Provincial Key Laboratory of Test Technology on Food Quality and Safety, Jinan 250100, China; 3 Institute of Sciences of Food Production, National Research Council, 70126 Bari, Italy; mariateresabrana@gmail.com (M.T.B.); miriam.haidukowski@ispa.cnr.it (M.H.); 4 Institute of Sciences of Food Production, National Research Council, 73100 Lecce, Italy; antonia.gallo@ispa.cnr.it (A.G.); antonio.logrieco@ispa.cnr.it (A.F.L.); 5 Oil Crops Research Institute, The Chinese Academy of Agricultural Sciences, Wuhan 430062, China; zhangqi01@caas.cn (Q.Z.); peiwuli@oilcrops.cn (P.L.)

**Keywords:** *Trichoderma*, biocontrol, *Aspergillus flavus*, aflatoxin, secondary metabolites

## Abstract

The inhibitory action of 20 antagonistic *Trichoderma* isolates against the aflatoxigenic isolate *A. flavus* ITEM 9 (Af-9) and their efficacy in reducing aflatoxin formation in vitro were examined. Production of metabolites with inhibitory effect by the *Trichoderma* isolates was also investigated. Antagonistic effect against Af-9 was assessed by inhibition of radial growth of the colonies and by fungal interactions in dual confrontation tests. A total of 8 out of 20 isolates resulted in a significant growth inhibition of 3-day-old cultures of Af-9, ranging from 13% to 65%. A total of 14 isolates reduced significantly the aflatoxin B_1_ (AfB_1_) content of 15-day-old Af-9 cultures; 4 were ineffective, and 2 increased AfB_1_. Reduction of AfB_1_ content was up to 84.9% and 71.1% in 7- and 15-day-old cultures, respectively. Since the inhibition of Af-9 growth by metabolites of *Trichoderma* was not necessarily associated with inhibition of AfB_1_ production and vice versa, we investigated the mechanism of reduction of AfB_1_ content at the molecular level by examining two strains: one (T60) that reduced both growth and mycotoxin content; and the other (T44) that reduced mycotoxin content but not Af-9 growth. The expression analyses for the two regulatory genes *aflR* and *aflS*, and the structural genes *aflA*, *aflD*, *aflO* and *aflQ* of the aflatoxin biosynthesis cluster indicated that neither strain was able to downregulate the aflatoxin synthesis, leading to the conclusion that the AfB_1_ content reduction by these *Trichoderma* strains was based on other mechanisms, such as enzyme degradation or complexation. Although further studies are envisaged to identify the metabolites involved in the biocontrol of *A. flavus* and prevention of aflatoxin accumulation, as well as for assessment of the efficacy under controlled and field conditions, *Trichoderma* spp. qualify as promising agents and possible alternative options to other biocontrol agents already in use.

## 1. Introduction

Aflatoxins are a group of potent mycotoxins produced by moulds of the genus *Aspergillus* in the course of spoilage of agricultural products, stored commodities, feeds and foods. Among the different forms of aflatoxins, aflatoxins B_1_, B_2_, G_1_ and G_2_ are especially important [1], since they exhibit carcinogenic, mutagenic and hepatotoxic effects [2]. The most potent form is aflatoxin B_1_ (AfB_1_), which is classified as carcinogenic to humans (group I) by the International Agency for Research on Cancer [3,4]. The main aflatoxigenic species is *Aspergillus flavus* [5], a saprophytic soil fungus with a large degree of genetic diversity, hundreds of different vegetative compatibility groups [6] and morphologically variable types, distinguished into two morphotypes based on sclerotia size, i.e., Group I (S strains) with sclerotia <400 μm in diameter and Group II (L strains) with sclerotia >400 μm in diameter [7]. L strains produce aflatoxins B_1_ and B_2_, are quite variable in the levels of aflatoxin produced and also include non-producing strains (non-aflatoxigenic strains). Conversely, S strains show less variation in aflatoxin production, are generally higher aflatoxin producers than L strains and can produce aflatoxins G_1_ and G_2_ in addition to aflatoxins B_1_ and B_2_ [8]. Another important aflatoxigenic species is *A. parasiticus*, which is able to produce all four of the above aflatoxins. *Aspergillus flavus* and *A. parasiticus* differ in host range and habitat. *Aspergillus flavus* occurs more widely on cereals, oilseeds and dried fruits, including economically important crops, such as peanuts and corn [9,10,11], while *A. parasiticus* is more strictly associated with soil environment and infections of belowground plant organs [12].

The occurrence of aflatoxins in foods and feeds is a major economic and food safety issue worldwide. Due to the danger to human and animal health, aflatoxins are highly regulated in most countries in the world, although regulations are not harmonized. To protect humans and animals from exposure to aflatoxins, the US Food and Drug Administration has set a limit of 20 μg/kg (ppb) of total aflatoxins in food and feed [13]; in China, according to the National Standards on Mycotoxins Limit in Food, the limits for AfB_1_ are 20 μg/kg in peanut, 10 μg/kg in rice and 5 μg/kg in wheat and related cereals [14]. In the European Union, the guidelines are more strict, setting a limit of 8 μg/kg AfB_1_ in food and 0.1 μg/kg AfB_1_ in cereal-based foods for babies and young children [15]. According to the Rapid Alert System for Food and Feed reports [16], in the last ten years AfB_1_ has been a frequent cause of rejection of imported products, leading to severe economic losses, especially for developing countries [17].

Aflatoxin-producing *Aspergillus* spp. generally infect plants in the field, leading to contamination of crops with aflatoxins at harvest and further aflatoxin accumulation under storage if temperature and humidity are not controlled properly [18]. Infections and aflatoxin contamination are more severe under plant-stressing climatic conditions, such as high heat and drought [19,20], thus the climate change and global warming are expected to increase the risk of aflatoxin occurrence in temperate areas in the years to come. In this scenario, management of aflatoxigenic fungi at pre-harvest becomes a fundamental part of a multiple action strategy to reduce the risk of aflatoxins at consumption [18]. Good cultural practices, such as balanced watering and fertilization, which reduce plant stress and injuries caused by pests, have mitigating effects on *A. flavus* infections and aflatoxin occurrence. However, these practices are not always possible or sufficient to lower the infections of *A. flavus* and aflatoxin formation to acceptable levels. The development of cultivars that are less subjected to *A. flavus* infections or aflatoxin biosynthesis still encounters major hurdles, including a lack of resistant genotypes in some crop species (e.g., cotton), the long time required for breeding programs and questionable stability of the resistance conferred by the currently available genes [21], while transgenic approaches are not universally accepted.

Studies on the genetic and aflatoxigenic diversity in *A. flavus* and on the dynamics of *A. flavus* populations of high and low aflatoxin-producing genotypes sharing the same niche have led to the development of a biological control strategy based on competitive exclusion [18,22]. The biocontrol relies on the inundative introduction of atoxigenic strains that are incapable of producing aflatoxins because they either lack necessary aflatoxin biosynthesis genes or have deleterious mutations in critical biosynthesis genes [23]. The atoxigenic *A. flavus* strains, once spread on the soil surface, with time displace the native aflatoxigenic genotypes by a mechanism of competition [22,24]. Currently, this strategy is being intensively studied and applied in an attempt to replace existing methods of chemical control, which may leave toxic residues or lead to development of resistant biotypes of the pathogen [25]. Nevertheless, the use of non-aflatoxigenic strains is not without drawbacks [26,27]. *Aspergillus flavus* is a heterothallic fungus, capable of sexual reproduction between individuals with different mating type loci (Mat1-1 and Mat1-2). Recombination can occur during sexual crosses and has also been detected between aflatoxigenic and non-aflatoxigenic strains. Therefore, the possibility exists that the capability to produce aflatoxins is transferred to the progeny of atoxigenic strains. In addition, other toxic metabolites on top of aflatoxins may be produced by *A. flavus*, including cyclopiazonic acid, aflatrem, aflavinines, paxillines, paspalicines and pseurotin [28,29]. These mycotoxins are not regulated and are not tracked by surveillance programs, but epidemiological data suggest that they might exert toxic effects on their own or act synergistically with aflatoxins [30,31]. Since the biosynthesis of those toxins is genetically regulated independently of aflatoxins, the non-aflatoxigenic biocontrol strains might still be capable of producing one or more of these other mycotoxins. Finally, it should be noted that, while the biocontrol *A. flavus* strains are non-aflatoxigenic, they are not necessarily non-pathogenic. In other words, they are able to reduce the level of aflatoxin contamination but might still cause severe crop disease and yield losses.

Apart from non-aflatoxigenic *A. flavus* strains, antagonistic microbes, including various bacteria [32,33,34,35,36,37,38,39,40] and yeasts [41,42,43,44], have shown remarkable inhibitory effects on the growth and/or on aflatoxin production of aflatoxigenic strains and have been considered as potential candidates for biocontrol of aflatoxin contamination at pre-harvest. Among the antagonists, fungi of the genus *Trichoderma* are possibly the most diffused biocontrol agents worldwide [45]. *Trichoderma* are free-living, mostly soil-resident, filamentous fungi that comprise rhizosphere-competent strains associated with root ecosystems [46]. Beneficial effects of *Trichoderma* include the ability to suppress both soil-borne and foliar plant pathogens, improvement of plant resistance to abiotic stresses and diseases, enhancement of mineral plant nutrition, promotion of plant growth and increase in crop productivity [47,48]. The outstanding success of *Trichoderma* spp. as biocontrol agents arises from their capability to exhibit multiple mechanisms of antagonistic action against plant pathogens [49]. Being fast-growing and metabolically flexible fungi able to use a variety of organic materials as food source, they are excellent competitors in soil environment. In addition, they produce a number of secondary metabolites inhibitory to plant pathogens [50], which function as chemical weapons within the so-called interference competition [51]. Although *Trichoderma* spp. are historically among the most studied agents for the biocontrol of soil pathogens, so far only few *Trichoderma* species and strains have been tested for biological control of *A. flavus* and aflatoxin production [52,53,54]. In this study we examined the inhibitory action of 20 antagonistic *Trichoderma* isolates against an aflatoxigenic strain of *A. flavus* and their effect on aflatoxin production, with an emphasis on the effect of Trichoderma metabolites and their involvement in the mode of action. In this regard, the effect of metabolites produced by two *Trichoderma* strains (namely *T.* *harzianum* T44 and *T.* *polysporum* T60) on the regulation of aflatoxin biosynthesis genes was investigated. In particular, we focused on the expression of the two regulatory genes *aflR* and *aflS* and four structural genes of the aflatoxin biosynthesis cluster, *aflA*, *aflD*, *aflO* and *aflQ*, by using the reverse transcriptase quantitative approach. Our results suggest that, unlike other microbial agents [55], *Trichoderma* are not able to downregulate the toxin biosynthesis, and aflatoxin reduction is based on other mechanisms, such as enzyme degradation or complexation.

## 2. Results

### 2.1. Antagonism of Trichoderma Isolates against A. flavus

**Figure 1:**
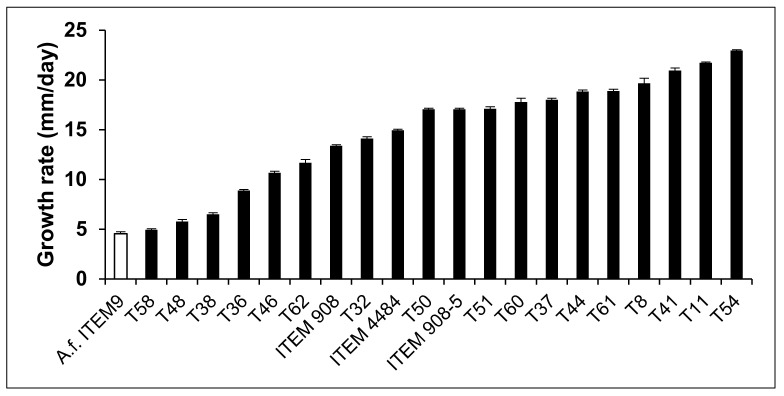
Average radial growth rate (mm/day) of *A. flavus* Af-9 and different *Trichoderma* isolates. Radial growth was measured daily in dual cultures for 3 days, until colony contact or until the arrest of growth if contact did not occur. Values are means ± SD (*n* = 3).

**Figure 2:**
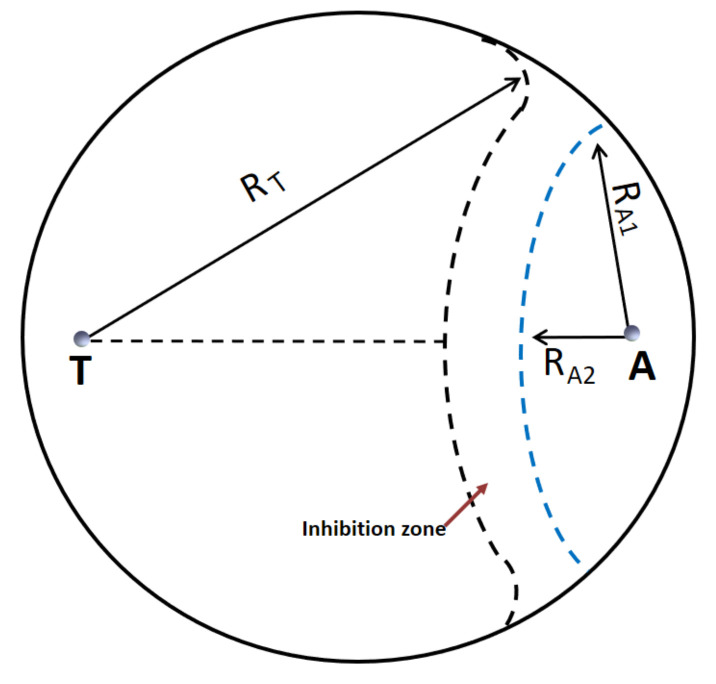
Representation of dual culture tests with *Trichoderma* spp. and *A. flavus* Af-9. **T** and **A** are the inoculation points of *Trichoderma* and Af-9, respectively. Undisturbed growth of either Af-9 or *Trichoderma* was assumed to be R_A1_ and R_T_, respectively. The percent inhibition of Af-9 radial growth was calculated as %I_DC_ = (R_A1_ − R_A2_)/R_A1_ × 100%.

**Figure 3:**
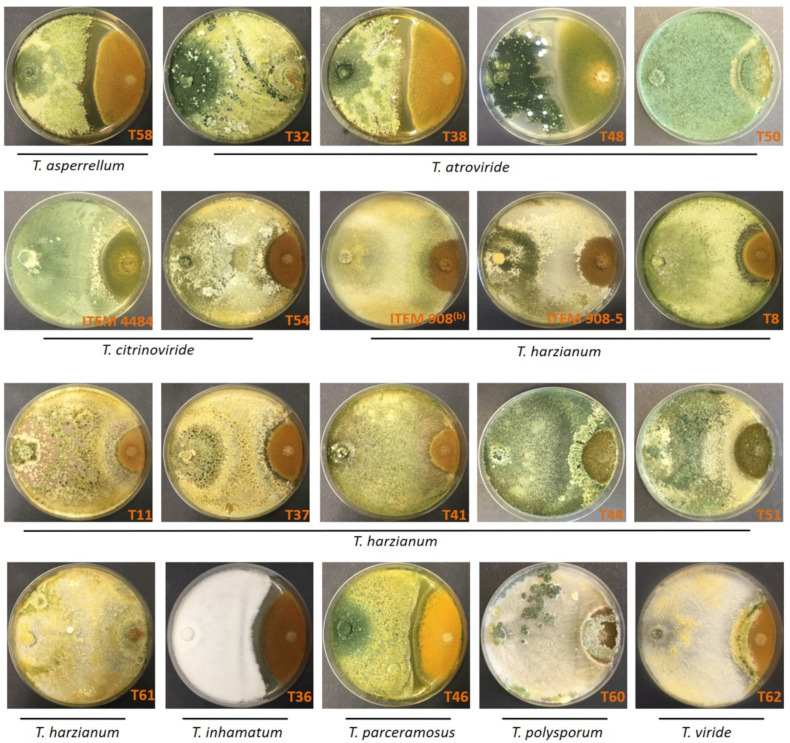
Confrontation test of *Trichoderma* spp. (on the left-hand side of the Petri dishes) and *A. flavus* Af-9 (on the right-hand side) colonies. Dual cultures on PDA after 21-day growth at 25 °C.

The average daily radial growth of *A. flavus* ITEM 9 (Af-9) was 4.6 mm/day; this is considerably slower than that of most of the *Trichoderma* isolates. The daily radial growth of *Trichoderma* colonies varied from 4.9 to 22.9 mm/day; that is up to three to four times faster than Af-9 (Figure 1). The fast-growing *Trichoderma* isolates rapidly spread and surrounded the colony of *A. flavus*, thus stopping its further enlargement. In several confrontations, the early contact between *Trichoderma* isolates and *A. flavus* resulted in the arrest of *A. flavus* growth before the differences between R_A1_ and R_A2_ became apparent (Figure 2 and Figure 3). As a result, in these confrontations, the value of percent inhibition of Af-9 colony growth was not statistically different from the control values (Table 1), in spite of the capability of *Trichoderma* to stop the growth of Af-9. More insight into the antagonistic potential of the *Trichoderma* isolates against *A. flavus* was obtained with the study of colony interactions, observed after 21 days of co-culture (Table 1, Figure 3). Overall, only type 1, 3 and 4 colony interactions were observed (Table 1); in none of the interactions have we observed overgrowth of *A. flavus* on *Trichoderma* spp. The *Trichoderma* isolates T32, T50, ITEM 908, T61, T60 and T62 arrested the growth of *A. flavus* after contact and aggressively overgrew the *A. flavus* colony (interaction type 1). The *Trichoderma* isolates T58, T38, T48, T8, T36 and T46 showed a different type of aggressive interaction with *A. flavus*, which resulted in a clear, ≥4 mm-wide inhibition zone (type 4). The rest of the strains (ITEM 4484, T54, ITEM 908-5, T11, T37, T41, T44, T51) showed a less aggressive interaction (type 3), with a mutual ≤2 mm-wide inhibition zone.

### 2.2. Inhibitory Effect of Trichoderma Metabolites on Growth of A. flavus

**Figure 4:**
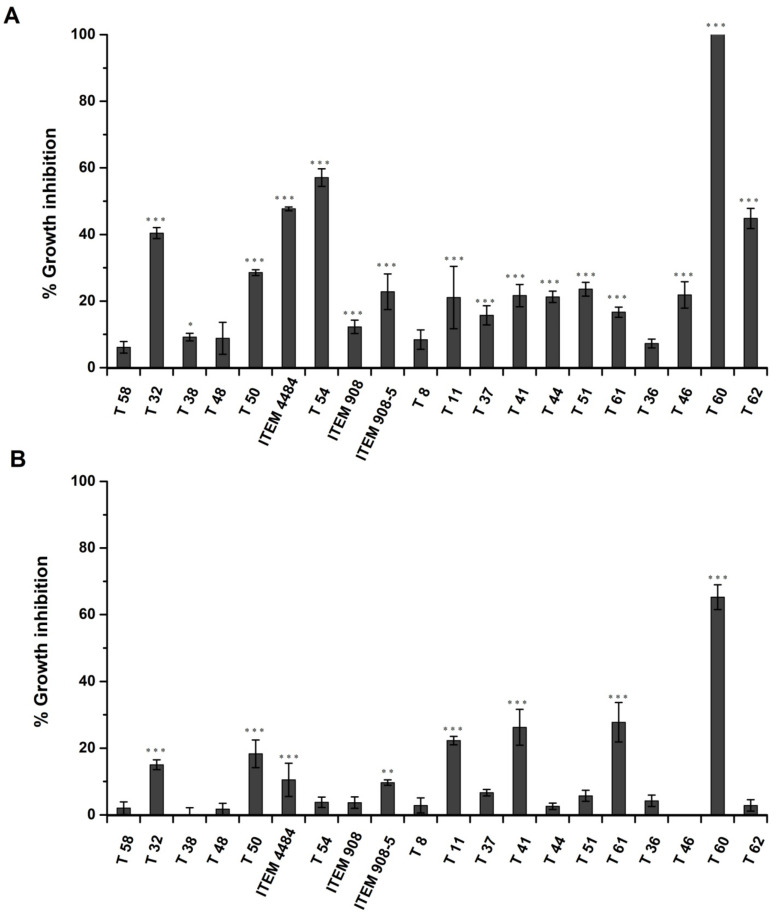
Inhibitory effect of *Trichoderma* spp. metabolites on the growth of *A. flavus* Af-9. The isolates of *Trichoderma* spp. were grown on sterilized cellophane sheets in Petri dishes containing Czapek Dox-Agar (**A**) or Czapek Dox-Agar supplemented with 2% peanut flour (**B**) (see text for more details) for 5 or 3 days, respectively. After removal of the cellophane sheet, the dishes were inoculated with Af-9 and incubated at 25 °C with 12/12 photoperiod for 6 days. Values are the means ± SD (*n* = 3) of the percent reduction of colony diameter with respect to control. Asterisks indicate statistically significant values at *p* < 0.05 (*), *p* < 0.01 (**) or = *p* < 0.001 (***) by one-way ANOVA.

Production of metabolites inhibitory to Af-9 by the 20 *Trichoderma* spp. isolates was investigated in both the standard Czapek Dox-Agar medium (CDA) and in Czapek Dox-Agar supplemented with 2% peanut flour (CDP). For all the isolates of *Trichoderma* tested, the inhibitory effect on growth of *A. flavus* was lower in CDP compared to CDA (Figure 4A,B). On CDA, 16 out of 20 isolates of *Trichoderma* significantly inhibited Af-9; in percentages, that ranged from 9 ± 1% to 100 ± 0% (Figure 4A). On CDP, only 8 out of 20 isolates resulted in a significant growth inhibition of Af-9; in percentages, that ranged from 13 ± 1% to 65 ± 3% (Figure 4B). All of them were also inhibitory on CDA. The most effective strains belonged to the species *T. atroviride* (T32 and T50), *T. citrinoviride* (ITEM 4484), *T. harzianum* species complex (ITEM 908-5, T11, T41, T61) and *T. polysporum* (T60). The strain T60 was the most effective in both media, resulting in 65 ± 3% and 100 ± 0% growth inhibition on CDP and CDA, respectively.

### 2.3. Inhibitory Effect of Trichoderma Metabolites on AfB_1_ Production by A. flavus

The inhibitory effect of *Trichoderma* metabolites on AfB_1_ production by Af-9 was initially investigated using both the media CDA and CDP. Analyses of AfB_1_ content in the media carried out after 7 and 15 days of growth showed that Af-9 was not able to produce AfB_1_ on CDA, regardless of the presence or absence of *Trichoderma* metabolites (data not shown). On the contrary, 7.3 ± 1.0 and 11.2 ± 2.6 μg/g of AfB_1_ were detected in control plates of CDP, respectively after 7- and 15-day growth of Af-9. Therefore, only CDP was used for the assessment of the inhibitory activity of *Trichoderma* metabolites on AfB_1_ production.

**Figure 5:**
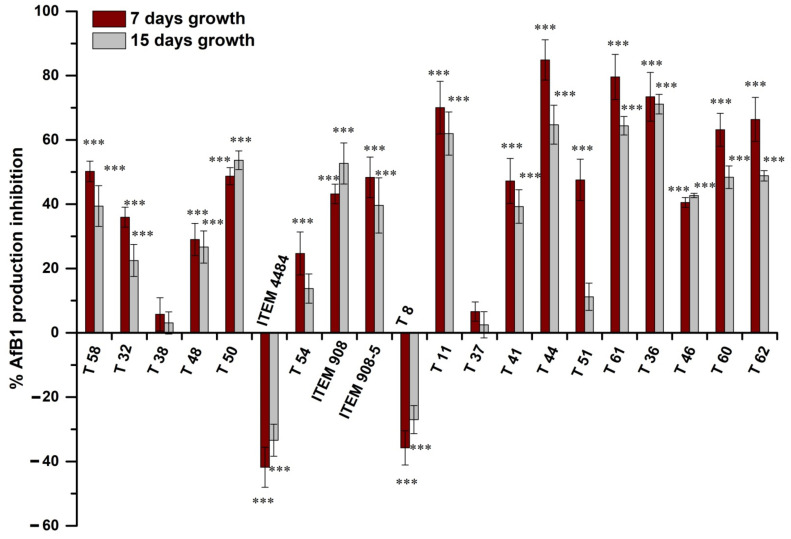
Percent reduction of AfB_1_ production by Af-9 grown for 7 and 15 days on CDP containing *Trichoderma* metabolites, with respect to control. In control plates, 7.3 ± 1.0 and 11.2 ± 2.6 μg/g of AfB_1_ were produced after 7 and 15 days, respectively. Values are the means ± SD of three replicates; statistically significant differences with control by one-way ANOVA are indicated by asterisks above the bar (*** = *p* < 0.001).

The inhibitory effect of *Trichoderma* isolates on AfB_1_ production is shown in Figure 5. Most *Trichoderma* isolates (14 out of 20) released metabolites in the medium that reduced significantly (*p* < 0.001) the production of AfB_1_, both at 7 and 15 days (Figure 5). In two cases (isolates T54 and T51), the inhibitory effect was temporary, since it was observed at 7 days but not at 15 days (Figure 5). Two isolates (T38 and T37) did not inhibit production of AfB_1_ either at 7 or 15 days (Figure 5). The reduction of AfB_1_ production by Af-9 ranged from 5.7 to 84.9% and from 2.5 to 71.1%, respectively, after 7 and 15 days. Interestingly, the isolates ITEM 4484 and T8 increased the production of AfB_1_ by Af-9, in spite of the former isolate being significantly inhibitory and the latter not being significantly inhibitory to Af-9 growth (Figure 4). The increase in AfB_1_ production was initially as high as 40% and then decreased to 30% at 15 days of growth (Figure 5).

### 2.4. Analysis of Aflatoxin Biosynthesis Gene Expression in Relation to the Control of Trichoderma

In order to investigate the molecular mechanism of the inhibitory effect exerted by *Trichoderma* metabolites on AfB_1_ production, the expression levels of aflatoxin biosynthesis genes were studied in Af-9 grown on CDP plates that were pre-inoculated with either *Trichoderma* isolate T60 or isolate T44. These two isolates were presumed to have different modes of inhibition of AfB_1_ production. Indeed, while T60 metabolites inhibited both mycelial growth and AfB_1_ production, T44 was very effective in inhibiting AfB_1_ production but did not significantly inhibit the growth of Af-9 (Figure 4 and Figure 5).

**Figure 6:**
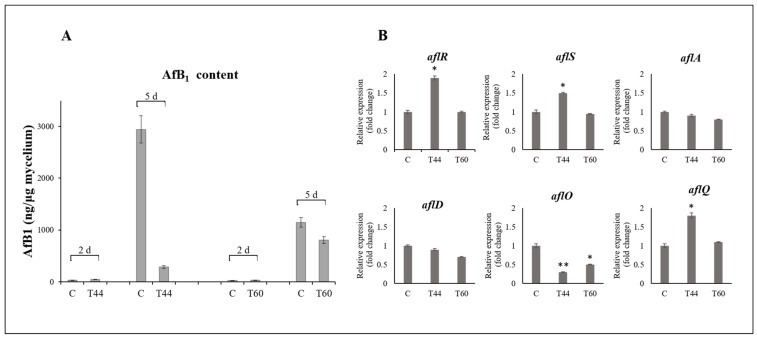
(**A**) Aflatoxin B_1_ content in *A. flavus* Af-9 mycelium after 2 and 5 days of growth on medium untreated (control, c) and pre-inoculated with *Trichoderma* strains T44 or T60; (**B**) expression analyses of aflatoxin biosynthesis genes in Af-9 after 2 days of growth on medium untreated (control, c) and pre-inoculated with *Trichoderma* strains T44 and T60. The *β-tubulin* gene was used as reference gene. Significantly differential gene expression is indicated (* *p*-value ≤ 0.05; ** *p*-value ≤ 0.01).

In this regard, the AfB_1_ production was determined per milligram of mycelium fresh weight so that, through the normalization of AfB_1_ content per unit of Af-9 biomass, the effect of reduction of AfB_1_ due to reduced growth was distinguishable from reduction due to the downregulation of biosynthesis. AfB_1_ was measured in the mycelium of Af-9 after 2 and 5 days of growth. As shown in Figure 6A, in the presence of T44 metabolites, a significant reduction (90.3%) of AfB_1_ production was observed after 5 days of growth. Conversely, when *A. flavus* was grown on plates pre-inoculated with T60, no significant difference was observed in the normalized production of AfB_1_ either at 2 or 5 days after inoculation (d.a.i.). The expression levels of the two regulatory genes (*aflR* and *aflS*) and four structural genes (a*flA*, a*flD*, *aflO* and *aflQ*) of the aflatoxin biosynthesis cluster were analyzed in Af-9 after 2 days of growth in control conditions or in plates pre-inoculated with the strain T44 or the strain T60. In Af-9 grown on CDP that was pre-inoculated with the isolate T44, the two regulatory genes *aflR* and *aflS* had a similar transcriptional trend, with a higher expression level than in control; on the contrary, no difference from control was observed when strain T60 was used (Figure 6B). A different transcriptional profile was observed among the structural genes (Figure 6B). In particular, for both the *Trichoderma* strains the expression levels of *aflA* and *aflD* showed no significant variation between control and treatment. On the other hand, the expression level of *aflO* decreased when Af-9 grew on medium pre-inoculated with either *Trichoderma* strains, more markedly with strain T44. By contrast, the expression level of *aflQ* increased significantly, as did that of the regulatory genes *aflR* and *aflS* when strain T44 was used in the pretreatment of the growth medium.

## 3. Discussion

Contamination of commodities with aflatoxins is a major issue for food safety and trade worldwide. Control of contamination at multiple points of the food chain, cultivation, transit, storage and processing, is necessary, but the control of infections by aflatoxigenic fungi at pre-harvest appears especially critical [11]. Spreading non-toxigenic *A. flavus* strains that competitively exclude aflatoxigenic strains in the field was first introduced by Cotty and Bayman [56], and this approach has proved to be successful in many cases, leading up to 80% reduction of aflatoxin contamination. It is currently utilized in cotton- and maize-growing areas of the USA and in Kenya and has led to the development of commercial biopesticides AF36 (non-aflatoxigenic strain NRRL 18543) and Afla-Guard^®^ (non-aflatoxigenic strain NRRL 21882) that are marketed in the USA for biocontrol of aflatoxins in groundnut and maize, respectively. However, some drawbacks of the method have been pointed out [26,27], which prompt the search for additional low-environmental-impact methods of control of aflatoxigenic fungi and aflatoxin in the field. *Trichoderma* spp. are well known and widely used biocontrol agents of root and foliar plant pathogens. Apart from the direct action against the pathogens, these beneficial fungi are also able to enhance plant mineral nutrition [47] and activate plant defenses and resistance against abiotic and biotic stresses [57,58], including drought [59] and herbivore insects [60,61]. Lately, a few research works reported on the capability of *Trichoderma* spp. to control *A. flavus* in the field, both in maize and peanut [62,63,64]. In the present research work, we have investigated the potential of 20 *Trichoderma* isolates belonging to different species for biological control of *A. flavus* and aflatoxins, with a focus on the effect of metabolites on *A. flavus* growth, AfB_1_ production and expression of genes of aflatoxin biosynthesis.

The confrontation test, also known as dual culture, is a method widely utilized for the in vitro selection of effective biocontrol strains of *Trichoderma* against various pathogens and has also been utilized for picking isolates antagonistic to *A. flavus* [65,66,67]. Usually, in this bioassay, the antagonistic capability of the biocontrol agent against a given pathogen is assessed by determination of the percent inhibition of colony radial growth of the challenged pathogen (%I). In our trials, we have observed that this parameter is not per se enough for a correct evaluation of the potential antagonistic capability of *Trichoderma* isolates against Af-9. Indeed, some aggressive and fast-growing *Trichoderma* isolates did not score high %I values, since the early contact between *Trichoderma* and Af-9 resulted in the arrest of pathogen growth before significant differences between the challenged colony growth and the unchallenged colony growth of Af-9 appeared. Therefore, it is very important that the %I value is carefully considered along with the type of colony interaction that occurs after the contact between the colonies, particularly the capability of the *Trichoderma* isolate to aggressively overgrow *A. flavus* (type 1 interaction).

Our experiments showed that in the antagonism of *Trichodema* spp. against Af-9, an important role is played by the production of metabolites. Metabolites produced by *Trichoderma* isolates and released in the growth medium were able to inhibit both the colony growth of Af-9 and its AfB_1_ production. However, it should be stressed that the effect of metabolites in inhibiting Af-9 growth was strongly affected by the medium used for the assessment. For all the isolates of *Trichoderma* tested, the inhibitory effect on *A. flavus* growth was higher in CDA compared to the same medium supplemented with 2% peanut flour (CDP). It is conceivable that some components of peanut flour either reduce the production of inhibitory metabolites by *Trichoderma* or enhance the *A. flavus* resistance. From a practical standpoint, this highlights the necessity to use media that contain natural components of the host plant for testing antagonistic efficacy of *Trichoderma* biocontrol isolates. Likewise, the presence of peanut flour in the medium enhanced the mycotoxigenicity of Af-9 and AfB_1_ biosynthesis, consistently with what had been observed in previous studies with other plant components [68,69]. It should also be noted that, for this experiment, “true” control plates were difficult to design. The pre-growth of *Trichoderma* on the medium that was subsequently exploited by Af-9 resulted in partial depletion of nutrients, which may have affected both Af-9 growth and AfB_1_ production. Conversely, depletion did not occur in control plates. Nevertheless, the experiment allowed us to assess the biocontrol capability of the different *Trichoderma* isolates comparatively. The inhibition of *A. flavus* colony growth by metabolites of *Trichoderma* was not necessarily associated with inhibition of AfB_1_ production and vice versa. Metabolites of the isolates T44 and T60 that were released in CDP proved to have a strong inhibitory effect on AfB_1_ production (approx. by 65–85% for T44 and 48–63% for T60, Figure 5). However, these two strains had a different effect on *A. flavus* growth (Figure 4). While T44 did not significantly inhibit mycelial growth, T60 had the highest inhibitory effect among all the strains tested (approx. by 65% on CDP). A similar differential effect on fungal growth and aflatoxin biosynthesis has been previously reported for some plant metabolites as well [70]. On this premise, it seemed interesting to investigate which inhibitory mechanism led to the reduction of AfB_1_ content, given the fact that only in the case of T60, but not in that of T44, could the lower amount of the mycotoxin be due to the reduction of Af-9 growth. Hence, the effect of metabolites of T44 and T60 on production of AfB_1_ was studied in correlation with the expression of some genes of aflatoxin biosynthesis cluster, in order to investigate the possible interference of *Trichoderma* metabolites at some points of the aflatoxin biosynthesis pathway. When the AfB_1_ content was analyzed in the 5-day-old mycelium of Af-9, the results confirmed that the reduction caused by metabolites of T60 was due to the inhibition of growth, while for metabolites T44, a different mechanism was conceivable. Recent studies have reported that the use of biocontrol agents or natural products can inhibit the production of AfB_1_ by downregulation of the genes involved in the biosynthesis of AfB_1_, even though the molecular mechanism behind this inhibition activity has not yet been completely determined [71]. For our study, we selected the two aflatoxin biosynthesis regulatory genes *aflR* and *aflS*, and the four structural genes, *aflA*, *aflD*, *aflO* and *aflQ*, for analysis of gene expression in 2-day-old mycelium of Af-9, since the regulation and expression of biosynthetic genes usually precede the production and accumulation of aflatoxin in the mycelium [72]. The *aflR* and *aflS* genes encode cluster-specific transcriptional factors that were suggested to interact to modulate and coordinate the expression of aflatoxin structural genes [73,74]. Among the selected structural genes, *aflA* and *aflD* act at the early phase of the biosynthesis pathway. The *aflA* gene encodes one of the two fatty acid synthases responsible for the first polyketide structure of the aflatoxin molecule, and the *aflD* gene encodes the enzyme responsible for the formation of the intermediate averantin. The other two genes *aflO* and *aflQ* are involved in the production of sterigmatocystin and hydroxyl-methylsterigmatocystin, the precursor of AfB_1_, respectively, acting in the later stages of the biosynthesis pathway [71]. From the analysis of expression levels in *A. flavus* grown in the presence of T60 metabolites, no difference was found compared to control. These findings were consistent with the results of AfB_1_ content, confirming that the T60 metabolites did not affect the biosynthetic mechanism of the mycotoxin. In the case of T44 metabolites, which led to a reduction in AfB_1_ production, the expression levels of most biosynthetic genes did not appear to be directly related to inhibitory activity. In fact, both the regulatory genes *aflR* and *aflS* and the structural gene *aflQ* showed an upregulation compared to the control. Conversely, the transcriptional levels of *aflA* and *aflD* genes remained unchanged, while only the *aflO* gene seemed downregulated compared to the control and apparently in line with the reduction in AfB_1_ production. Usually, the inhibitory activity exerted by biological control agents on the AfB_1_ biosynthesis by *A. flavus* is correlated with the downregulation of all or most of the approximately 27 genes composing the aflatoxin biosynthesis cluster [55,75]. In our study, we observed an increase in the expression of *aflR* and *aflS* in the presence of T44 metabolites, and only the *aflO* was downregulated. Further analyses are necessary to examine whether the expression of other genes of the biosynthesis cluster may be affected and establish whether the negative regulation of AfB_1_ production occurs at the biosynthesis cluster level or during translational and/or post-translational stages, causing low levels of proteins or lack of functionality. It cannot be excluded that some of the metabolites produced by strain T44 could activate a signaling pathway, leading to the upregulation of regulatory genes *aflR* and *aflS* of the aflatoxin cluster. On the other hand, the observed reduction in aflatoxin accumulation in the culture media could be due to mechanisms triggered by other *Trichoderma* metabolites. Several works have reported the ability of microorganisms, including *Trichoderma* species, to produce enzymes capable of degrading or modifying mycotoxin molecules [76]. Finally, it should be considered that in confrontation tests, 2 out of the 20 *Trichoderma* strains tested, namely ITEM 4484 and T8, resulted in an augmented capability of Af-9 to produce AfB_1_ (Figure 5), even if the antagonists significantly reduced Af-9 growth. While upregulation of AfB_1_ biosynthesis genes cannot be ruled out, other hypotheses, including digestion of medium components by Trichoderma enzymes, which generate compounds boosting AfB_1_ production, also deserve further investigation.

The option of using *Trichoderma* spp. for biocontrol of aflatoxigenic fungi may offer a few advantages in respect to the use of non-aflatoxigenic *A. flavus* strains. First, the non-aflatoxigenic *A. flavus* strains are not necessarily non-pathogenic, and they might still cause disease and yield loss. As broad-spectrum biocontrol agents, *Trichoderma* may also protect the plants from the attack of other plant pathogens in addition to *A. flavus* [58,77,78]. Finally, the indirect action of *Trichoderma* on the enhancement of plant resilience to drought stress and on the prevention of insect pest damages, both of which are factors facilitating aflatoxin occurrence [79], is one more point for consideration of *Trichoderma* in the context of *A. flavus* control and prevention of aflatoxin occurrence.

## 4. Conclusions

In conclusion, the results reported herein have shown the potential of isolates belonging to different species of *Trichoderma* to be biocontrol agents for *A. flavus* and prevent aflatoxin accumulation. Both of these properties may be heavily influenced by the presence of host-plant-derived components in the growth medium; therefore, the *in vitro* selection of effective strains for further assessment should be compiled, taking into account the crop on which they will be used. The biocontrol *Trichoderma* strains may arrest fungal growth, reduce aflatoxin production or both. Metabolites of *Trichoderma* spp. play a role in their mechanism of action, although with a diversity of modes. Some are inhibitory to *A. flavus* growth; others reduce the accumulation of aflatoxin, presumably via degradation. Thus, an effective biocontrol strategy may be based on the combined use of multiple isolates with different mechanisms of action. Although further studies are envisaged to identify the metabolites involved in the biocontrol of *A. flavus* and prevention of aflatoxin accumulation, as well as for assessment of the efficacy under controlled and field conditions, *Trichoderma* spp., including a few strains tested within the present work, qualify as promising agents and possible alternative options to other biocontrol agents already in use.

## 5. Materials and Methods

### 5.1. Fungal Strains

The isolates of *Trichoderma* spp. used in this study were collected from soil or plant debris, or obtained from the culture collection of the Institute of Science of Food Production (ITEM Collection, http://www.ispa.cnr.it/Collection/ (accessed on 2 December 2021), Bari, Italy) (Table 2). All the isolates were identified morphologically, according to Gams and Bisset [80]. The commercial biocontrol strain *T. atrobrunneum* ITEM 908 (*T. harzianum* species complex, formerly *T. harzianum* ITEM 908) was previously molecularly characterized [81] by analysis of the sequences of the internal transcribed spacer regions ITS-1 and ITS-2 of the nuclear rDNA and of a fragment of the translation elongation factor gene TEF-1α, according to Chaverri et.al. [82]. The mutant strain ITEM 908-5 was generated by UV irradiation of the parental strain ITEM 908 [83]. The AfB_1_-producing strain *Aspergillus flavus* ITEM 9 (Af-9 = NRRL 3251, ATCC 36061) was used for all the tests throughout the work. Af-9 was originally isolated from walnuts in the USA and is a high AfB_1_-producing strain [84]. Fungal cultures were maintained in purity on potato dextrose agar (PDA, Oxoid, Italy) slants at + 5 °C, which were used for preparation of fresh cultures and inocula.

### 5.2. Antagonism of Trichoderma *spp.* against A. flavus

The antagonism and colony interaction between each *Trichoderma* spp. isolate and Af-9 were studied in vitro by dual cultures. Petri dishes of 9 cm in diameter containing 20 mL of PDA were inoculated 1 cm apart from the edge of the plate with a 6 mm-diameter mycelial plug from a fresh culture of *Trichoderma* and on the opposite side, at a 7 cm distance from the *Trichoderma* inoculation point, with a 10 μL drop of spore suspension of Af-9 containing 1 × 10^7^ conidia/mL in sterile distilled water. All the pathogen–antagonist co-cultures were incubated at 25 °C ± 1 with 12/12 photoperiod. The radial growth of both the colonies was measured daily until contact or until the arrest of colony growth if contact did not occur because of mutual inhibition, and the average daily radial growth (mm/day) of each fungus was calculated. The percent inhibition of Af-9 radial growth in dual cultures (%I_DC_) was calculated as

%I_DC_ = (R_A1_ − R_A2_)/R_A1_ × 100 (1)

where R_A1_ was the longest radius of the Af-9 colony, and R_A2_ was the radius of the Af-9 colony along the line that connected the Af-9 and the *Trichoderma* inoculation points (Figure 2), measured on the day of contact or the last day of incremental growth, if contact did not occur.

At 21 days after inoculation (d.a.i.), the colony interactions were assessed visually and classified in accordance with Whipps [52] as: 1 = *Trichoderma* overgrowing Af-9 and Af-9 stopped; 1/2 = *Trichoderma* overgrowing Af-9 but Af-9 still growing; 2/1 = Af-9 overgrowing *Trichoderma* but *Trichoderma* still growing; 2 = Af-9 overgrowing *Trichoderma* and *Trichoderma* stopped; 3 = slight mutual inhibition (inhibition zone ≤ 2 mm-wide); 4 = strong mutual inhibition (inhibition zone ≤ 4 mm-wide). The experiment was carried out in triplicate.

### 5.3. Effect of Non-Volatile Metabolites of Trichoderma *spp.* on A. flavus Growth

The inhibitory effect of *Trichoderma* metabolites on the growth of *A. flavus* was studied in Czapek Dox-Agar (CDA, Biolife Italiana, Monza, Italy) and Czapek Dox-Agar supplemented with 2% (w/v) peanut flour (CDP). For preparation of CDP, peanuts were finely ground in a laboratory mill (Mulino Cyclone, International PBI, Milano, Italy) to particles of <0.2 mm; 0.4 g of ground peanuts were transferred into 2.5 cm-diameter and 15 cm long test tubes that were filled with 20 mL of melted CDA and autoclaved at 121 °C for 15 min. After cooling at 55 °C, the medium was thoroughly mixed by a vortex mixer and quickly poured into 9 cm-diameter Petri dishes. After solidification of the medium, a pre-autoclaved (121 °C, 15 min) cellophane disc was laid on the medium to cover the entire surface. Mycelial plugs of 6 mm in diameter were removed from the edge of 5-day-old cultures of *Trichoderma* and transferred to the center of the Petri dishes, onto the cellophane disc. The cellophane membrane was used to keep the mycelium from invading the medium, at the same allowing time the metabolites to diffuse into the substrate through the cellophane pores. The colonies of *Trichoderma* were grown for 5 days on CDA and for 3 days on CDP, at 25 ± 1 °C with 12/12 photoperiod; then, the cellophane sheets with the overlying *Trichoderma* colonies were removed. The plates were center-inoculated with a 10 μL drop of a 1 × 10^7^ conidia/mL spore suspension of Af-9 and incubated at 25 ± 1 °C with 12/12 photoperiod for 6 days. Control plates were prepared by inoculating Af-9 on CDA and CDP without previous cultivation of *Trichoderma*. The experiment was carried out in triplicate. The growth of *A. flavus* was assessed by the colony diameter, measured with a ruler under a dissecting microscope every 24 h. The percent inhibition of the diametral growth of Af-9 colonies (%I_D_) caused by *Trichoderma* metabolites was calculated with the following formula

%I_D_ = (D_C_ − D_T_)/D_C_ × 100 (2)

where D_T_ was the average diameter of Af-9 colonies grown on media pre-inoculated with *Trichoderma*, and D_C_ was the average diameter of Af-9 colonies grown in control plates.

### 5.4. Determination of AfB_1_ by UPLC

AfB_1_ was determined by Ultra Performance Liquid Chromatography (UPLC) using the Acquity UPLC system (Waters, Milford, MA, USA). Data acquisition and instrument control were performed by Empower 2 software (Waters). The column used was a 100 mm × 2.1 mm i.d., 1.7 μm, Acquity UPLC1 BEH RP-18, with an Acquity UPLC column in-line filter (0.2 μm), detected by fluorometric detector without post-column derivatization. The fluorometric detector was set at wavelengths of 365 nm (excitation) and 435 nm (emission). The mobile phase was a mixture of water–acetonitrile–methanol (64:18:18, *v*/*v*/*v*) at a flow rate of 0.4 mL/min. The temperature of the column was maintained at 40 °C. In these experimental conditions, the retention time of the AfB_1_ standard was 3.7 min (see Supplementary Figure S1). AfB_1_ was quantified by measuring the peak areas of the samples at the retention time of the aflatoxin standard and comparing these areas with the calibration curve of AfB_1_ in the range of 0.2 to 10.0 ng/mL. The limit of quantification (LOQ) of the method was 0.2 ng/mL for AfB_1_, based on a signal to noise ratio of 10:1.

### 5.5. Effect of Trichoderma Metabolites on Aflatoxin Production by A. flavus

Media, inoculations and controls were made in the same way as described for the experiment on the effect of *Trichoderma* non-volatile metabolites on the growth of Af-9. Each *Trichoderma* isolate was tested in three replicates. After 7- and 15-day growth of *A. flavus*, the cultures were sampled for the determination of AfB_1_. An 8 mm-diameter cork-borer was used to excise mycelial discs at regular distances along the radius of the colony, starting from the inoculation point to the edge of the colony. Excised discs were then transferred to test tubes. The samples (approximately 1 g) were precisely weighted and extracted with 5 mL of a methanol–water (80: 20, *v*/*v*) solution in a KS 4000i orbital shaker (IKA Werke GmbH & Co. KG., Staufen, Germany) at 250 rpm for 60 min at room temperature. Samples were then centrifuged, filtered and diluted to obtain extracts, which were stored at −20 °C until AfB_1_ analysis, according to previously described methods [85,86]. The inhibition rate of AfB_1_ production (%I_AfB1_) was calculated with the following formula

%I_AfB1_ = (AfB_1C_ − AfB_1T_)/AfB_1C_ × 100 (3)

where AfB_1T_ was the amount of AfB_1_ produced by *A. flavus* on medium pre-inoculated with *Trichoderma*, and AfB_1C_ was the amount of AfB_1_ in the control plates.

### 5.6. Effect of Metabolites of Trichoderma on the Expression of Aflatoxin Biosynthesis Genes

#### 5.6.1. Strains Culture and Sampling of Mycelia

For this study, the *Trichoderma* strains T44 and T60 and Af-9 were grown on CDP. Preparation of CDP and cultivation of the fungi on cellophane sheets were conducted as described in Section 5.3. After 3 days of growth, the membrane and the colony of *Trichoderma* were removed from the Petri dish. Then, the medium was inoculated with 100 μL of a spore suspension of Af-9 containing 1 × 10^7^ spores/mL, which were spread evenly with an L-shaped spreader. As controls, Af-9 was inoculated on cellophane sheets laid on CDP not pre-inoculated with *Trichoderma*. Cultures of *A. flavus* were incubated at 25 ± 1 °C with 12/12 photoperiod, and the mycelium was collected by scraping it off the cellophane at two time points, i.e., 2 and 5 d.a.i. The mycelium samples were stored at −20 °C until extraction and AfB_1_ analysis, according to the procedure described in Section 5.4. For the 2 d.a.i. samples, the cellophane sheets were cut in half, and the mycelium grown in each half was scraped off, weighted, immediately frozen in liquid nitrogen and stored at −80 °C. The mycelium from one half plate was used for RNA extraction, while the mycelium from the other half plate was used for AfB_1_ analysis. At each time point, analyses were performed using the pooled mycelium from 10 plates.

#### 5.6.2. RNA Extraction and cDNA Synthesis

Total RNA was extracted using the RNeasy kit (Qiagen, Valencia, CA, USA), and an on-column DNase I treatment was performed, according to the manufacturer’s protocols. The A_260_/A_280_ ratio was determined using a NanoDrop 1000 (Thermo Fisher Scientific, Waltham, MA, USA), and gel electrophoresis was performed for qualitative analysis of total RNA. The cDNA was synthesized using 2.0 μg total RNA, oligo (dT) 18 primer and random hexamers, and SuperScript III Reverse Transcriptase (Invitrogen, San Diego, CA, USA), according to instructions of the manufacturer.

#### 5.6.3. Expression Analysis of Aflatoxin Biosynthesis Gene by Quantitative Reverse Transcriptase PCR (RT-qPCR)

The StepOne™ Real-Time PCR System (Applied Biosystem, Waltham, MA, USA) was used to carry out RT-qPCR assays. The transcription profiles of 6 genes of the aflatoxin biosynthesis cluster (*aflR*, *aflS*, *aflA*, *aflD*, *aflO*, *aflQ*) were analyzed, and the housekeeping *β-tubulin* gene was used as reference gene. Sequences of primers listed in Table 3 were retrieved from previous publications [55,72]. The sequences of all genes were obtained from the *A. flavus* NRRL3557 genome database at NCBI (BioProject PRJNA575750). Each reaction was performed in a total volume of 20 µL containing cDNA (3.8 ng/µL), 12.5 μL of SYBR Green Supermix (Bio-Rad, Hercules, CA, USA) and different concentrations of primers (Table 3). Nuclease-free water was added to make the total volume 20 uL. The PCR reaction consisted of initial denaturation at 95 °C for 5 min, 40 cycles at 94 °C for 30 s, 57 °C for 30 s and 72 °C for 15 s.

The relative quantification of gene expression was established by using the 2^−ΔΔCt^ method [87]. Statistical analysis was performed by using the *t*-test.

### 5.7. Chemicals and Reagents

The chemical standard of AfB_1_ (purity > 99%) was supplied by Sigma-Aldrich (Milan, Italy). All solvents (HPLC grade) were purchased from VWR International Srl (Milan, Italy). The water Millipore Milli-Q system was purchased from Millipore (Bedford, MA, USA). Immunoaffinity columns (Aflatest^®^ WB) were obtained from Vicam (Watertown, MA, USA). Glass microfiber filters (Whatman GF/A), paper filters (Whatman no. 4) and regenerated cellulose membrane filters (RC, 0.2 mm) were obtained from Grace (Deerfield, IL, USA).

### 5.8. Statistical Analysis

Data were analyzed by one-way analysis of variance (ANOVA) and Tukey–Kramer multiple comparison test. The statistical analyses were performed using the GraphPad Instat 3.0 software (GraphPad Software, San Diego, CA, USA). Figures were drawn using OriginPro9.0 software (OriginLab Corporation, Northampton, MA, USA).

## Figures and Tables

**Table 1:** Inhibition of the aflatoxigenic isolate Af-9 growth by *Trichoderma* spp. and type of colony interactions in confrontation tests (dual cultures) on PDA.

Antagonistic Species/Strain	%I_DC_^(a)^	*p* Value ^(b)^	Interaction Type ^(c)^
*Trichoderma* *asperellum*			
T58	35.6 ± 3.45	**	4
*Trichoderma* *atroviride*			
T32	2.88 ± 0.98	n.s.	1
T38	20.0 ± 3.18	*	4
T48	42.3 ± 3.21	**	4
T50	0.00 ± 0.00	n.s.	1
*Trichoderma citrinoviride*			
ITEM 4484	4.45 ± 1.93	n.s.	3
T54	1.90 ± 1.65	n.s.	3
*Trichodermaharzianum* species complex (incl. *T. atrobrunneum*)			
ITEM 908	12.5 ± 2.43	**	1
ITEM 908-5	5.75 ± 2.00	n.s.	3
T8	5.24 ± 1.75	n.s.	4
T11	0.00 ± 0.00	n.s.	3
T37	0.00 ± 0.00	n.s.	3
T41	0.00 ± 0.00	n.s.	3
T44	0.00 ± 0.00	n.s.	3
T51	5.24 ± 1.75	n.s.	3
T61	1.15 ± 1.20	n.s.	1
*Trichoderma inhamatum*			
T36	37.9 ± 0.01	**	4
*Trichoderma* *parceramosus*			
T46	21.2 ± 0.85	**	4
*Trichoderma* *polysporum*			
T60	2.30 ± 1.99	n.s.	1
*Trichoderma* *viride*			
T62	3.70 ± 1.60	n.s.	1

^(a)^ Percent inhibition of radial growth of *A. flavus* colonies; means ± SD (*n* = 3). ^(b)^ Asterisks indicate statistically significant differences from control values by one-way ANOVA. ** = *p* < 0.01; * = *p* < 0.05, n.s. = not significant. ^(c)^ Interaction type, modified from Whipps (1987) [52]: 1 = *Trichoderma* overgrowing *A. flavus* and *A. flavus* stopped; 1/2 = *Trichoderma* overgrowing *A. flavus* but *A. flavus* still growing; 2/1 = *A. flavus* overgrowing *Trichoderma* but *Trichoderma* still growing; 2 = *A. flavus* overgrowing *Trichoderma* and *Trichoderma* stopped; 3 = slight mutual inhibition (inhibition zone ≤ 2 mm-wide); 4 = strong mutual inhibition (inhibition zone ≤ 4 mm-wide).

**Table 2:** Strains of *Trichoderma* spp. used in this study.

Species/Strain ^(a)^	Geographical Origin	Source
*Trichoderma asperrellum*		
T58	Not known	Not known
*Trichoderma atroviride*		
T32	Not known	Not known
T38	Not known	Not known
T48	Italy	Soil
T50	USA	Corn kernel
*Trichoderma citrinoviride*		
ITEM 4484	Austria	Forest soil
T54	Not known	Maize
*Trichoderma harzianum* species complex (incl. *T. atrobrunneum*)		
ITEM 908 ^(b)^	Italy	Olive
ITEM 908-5	-	UV-mutant of ITEM 908
T8	Italy	Mushroom substrate
T11	Italy	Corn kernel
T37	Italy	Seedling soil mix
T41	Borneo, Asia	Soil
T44	Italy	Mushroom substrate
T51	USA	Corn kernel
T61	Not known	Not known
*Trichoderma inhamatum*		
T36	Not known	Not known
*Trichoderma parceramosus*		
T46	Italy	Mushroom substrate
*Trichoderma polysporum*		
T60	Italy	Chestnut soil
*Trichoderma viride*		
T62	Italy	Eggplant leaf

^(a)^ Morphological species determined according to Gams and Bisset [80]. The strains with an ITEM number are from the culture collection of the Institute of Sciences of Food Production (http://www.ispa.cnr.it/Collection/ (accessed on 2 December 2021)). ^(b)^ Identified as *T. atrobrunneum* by sequence analysis of ITS-1, ITS-2 and TEF-1α, as reported by Fanelli et al., 2018 [81].

**Table 3:** Primers used in this study.

Gene	Primer Code	Concentration	Sequence (5′–3′)	Fragment Length
*β-tubulin* ID 64852080	AFtub_for	100 nM	GGTCGTTACCTCACCTGCTCT	79 bp
	AFtub_rev		GGATGTTGCGCATCTGGT	
*aflR* ID 64848036	aflR_for	100 nM	CGGCACAGCTTGTTCTGAGT	88 bp
	aflR_rev		GCATCGTCTCCACCTTCTTG	
*aflS* ID 64848035	aflS_for	150 nM	CTGGCAAAACTTGGGAATGG	103 bp
	aflS_rev		CACGAGGAAACGGAGTGATG	
*aflA* ID 64848038	aflA_for	250 nM	CATGCTGTTAACCCCCGACT	111 bp
	aflA_rev		AATTGGGCTAGGAAACCGGG	
*aflD* ID 64848039	aflD_for	100 nM	GCGCAAGTTCCACTTTGAGA	84 bp
	aflD_rev		CCTTGGTCGCCCATATCAGT	
*aflO* ID 64848026	aflO_for	100 nM	GTGCGGTGGTGCAACTATTC	71 bp
	aflO_rev		TCTCTCGGCCAGGAAGTCA	
*aflQ* ID 64848029	aflQ_for	250 nM	GCACCAACAATTCGGCTCTG	134 bp
	aflQ_rev		TGTGGAAGGGTGGAAGATGC	

## Data Availability

The data presented in this study are available on request from the corresponding author.

## Supplementary Materials

The following supporting information can be downloaded at: https://www.mdpi.com/article/10.3390/toxins14020086/s1, Figure S1: Chromatograms of aflatoxin B1 (AFB1) and B2 (AFB2) standards A) 5.0 ng/mL of AFB1 and 1 ng/mL AFB2 and B) 0.4 ng/mL of AFB1 and 0.08 ng/mL AFB2 in UPLC/PDA; Chromatograms of the diluted mycelium extract from the samples of Af-9 grown on CDP containing C) metabolites of T44 (AFB1 0.04 µg/g) and D) T60 (AFB1 0.02 µg/g).
