# Supplementary material for: Potential of Trichoderma spp. for Biocontrol of Aflatoxin-Producing Aspergillus flavus
Source: Toxins (Basel). 2022 Jan 23;14(2):86. doi: 10.3390/toxins14020086 (PMC8875375; doi:10.3390/toxins14020086)
Supplement: Supplementary file 1 [file toxins-14-00086-s001.zip › toxins-1546009-supplementary.pdf]

toxins

MDPI

Article

Potential of *Trichoderma* spp. for Biocontrol of Aflatoxin-Producing *Aspergillus flavus*

Xianfeng Ren, Maria Teresa Branà, Miriam Haidukowski, Antonia Gallo, Qi Zhang, Antonio F. Logrieco, Peiwu Li, Shancang Zhao and Claudio Altomare

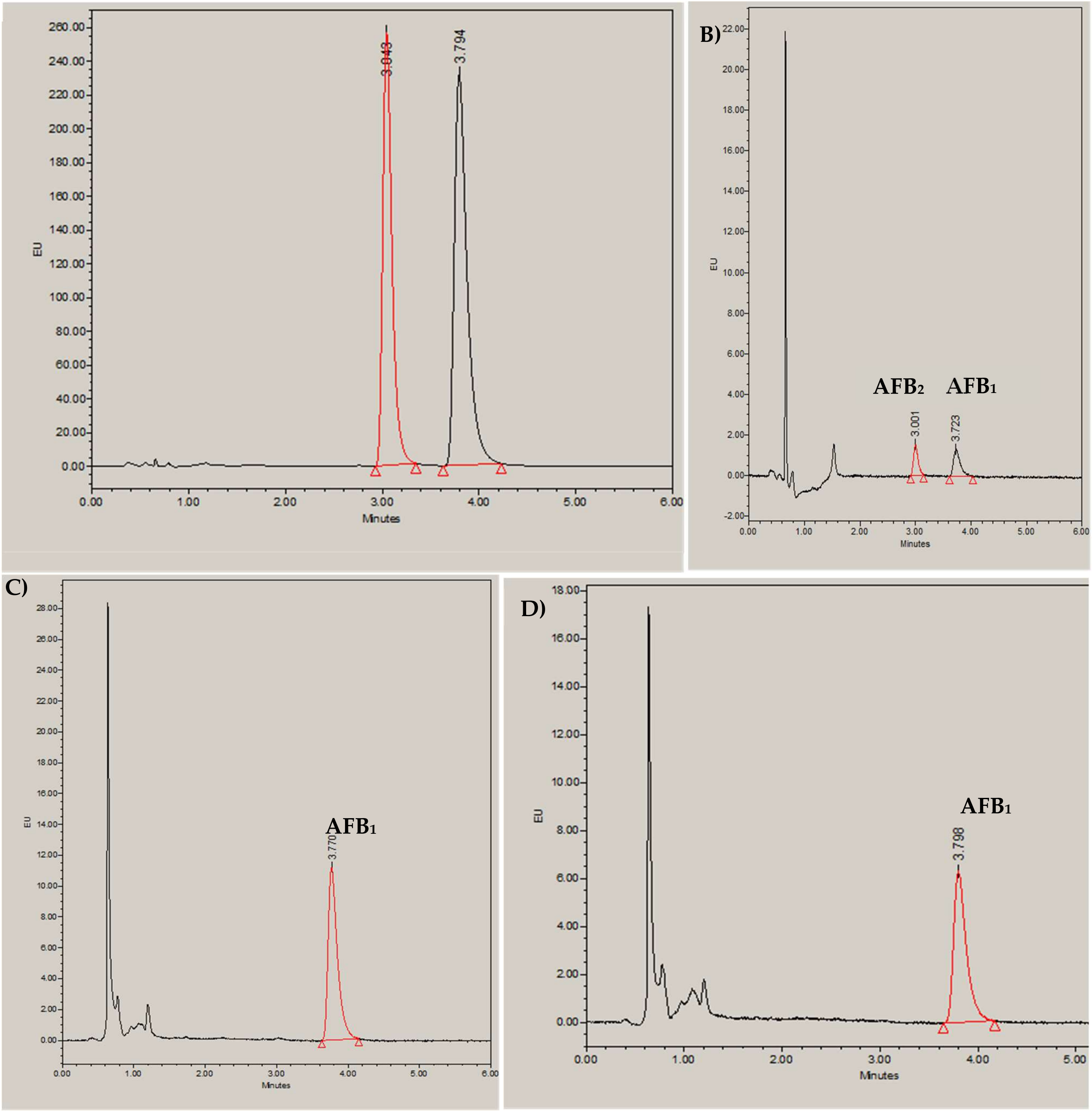

Figure S1. Chromatograms of aflatoxin B_1 (AFB_1) and B_2 (AFB_2) standards (**A**) 5.0 ng/mL of AFB_1 and 1 ng/mL AFB_2 and (**B**) 0.4 ng/mL of AFB_1 and 0.08 ng/mL AFB_2 in UPLC/PDA; Chromatograms of the diluted mycelium extract from the samples of Af-9 grown on CDP containing (**C**) metabolites of T44 (AFB_1 0.04 μg/g) and (**D**) T60 (AFB_1 0.02 μg/g).
